# Supplementary material for: Neuroanatomical Correlates of Semantic Features of Narrative Speech in Semantic and Logopenic Variants of Primary Progressive Aphasia
Source: Brain Sci. 2022 Jul 12;12(7):910. doi: 10.3390/brainsci12070910 (PMC9320086; doi:10.3390/brainsci12070910)
Supplement: Supplementary file 1 [file brainsci-12-00910-s001.zip › brainsci-1796675-supplementary.pdf]

Table S1. Means and standard deviations on cortical thickness of the three groups (svPPA, lvPPA, HC) and comparisons performed between each clinical group and Heathy Controls (HC) with Mann-Whitney U Tests. Abbreviations: SD = standard deviation; svPPA = semantic variant of Primary Progressive Aphasia; lvPPA = logopenic variant of Primary Progressive Aphasia. P-values were adjusted using the False Discovery Rate – (FDR) correction (Benjamini & Hochberg, 1995). In bold are reported statistical significant results.

	svPPA		lvPPA		HC		Pairwise comparisons (pFDR)	
	Mean	SD	Mean	SD	Mean	SD	svPPA vs HC	lvPPA vs HC
lh bankssts thickness	1.87	0.31	1.98	0.24	2.35	0.17	**0.005**	**< 0.001**
lh caudalanteriorcingulate thickness	2.61	0.19	2.58	0.37	2.68	0.29	0.390	0.782
lh caudalmiddlefrontal thickness	2.14	0.26	2.05	0.25	2.40	0.16	**0.018**	**< 0.001**
lh cuneus thickness	1.62	0.15	1.68	0.18	1.81	0.18	**0.022**	**0.021**
lh entorhinal thickness	1.77	0.27	2.60	0.51	3.20	0.42	**< 0.001**	**< 0.001**
lh fusiform thickness	1.99	0.25	2.18	0.43	2.56	0.11	**< 0.001**	**< 0.001**
lh inferiorparietal thickness	1.93	0.27	1.91	0.28	2.29	0.13	**0.005**	**< 0.001**
lh inferiortemporal thickness	1.99	0.31	2.30	0.37	2.66	0.19	**< 0.001**	**< 0.001**
lh isthmuscingulate thickness	2.01	0.11	1.97	0.27	2.29	0.24	**0.010**	**0.002**
lh lateraloccipital thickness	1.94	0.15	1.86	0.21	2.01	0.15	0.179	**0.008**
lh lateralorbitofrontal thickness	2.27	0.30	2.50	0.34	2.57	0.15	**0.023**	0.981
lh lingual thickness	1.80	0.13	1.75	0.22	1.91	0.16	0.104	0.017
lh medialorbitofrontal thickness	2.12	0.20	2.35	0.36	2.40	0.13	**0.008**	0.917
lh middletemporal thickness	2.04	0.33	2.34	0.35	2.70	0.18	**< 0.001**	**< 0.001**
lh parahippocampal thickness	1.88	0.24	2.25	0.46	2.58	0.26	**< 0.001**	**0.005**
lh paracentral thickness	2.05	0.30	1.96	0.32	2.23	0.25	0.104	**0.004**
lh parsopercularis thickness	2.22	0.26	2.21	0.24	2.45	0.13	**0.040**	**< 0.001**
lh parsorbitalis thickness	2.34	0.35	2.43	0.32	2.54	0.14	0.199	0.476
lh parstriangularis thickness	2.13	0.28	2.12	0.27	2.30	0.13	0.155	**0.025**
lh pericalcarine thickness	1.54	0.17	1.51	0.17	1.55	0.20	0.966	0.662
lh postcentral thickness	1.78	0.13	1.72	0.17	2.00	0.15	**0.008**	**< 0.001**
lh posteriorcingulate thickness	2.09	0.24	2.11	0.34	2.40	0.20	**0.017**	**0.005**
lh precentral thickness	2.13	0.30	2.04	0.30	2.38	0.24	0.063	**< 0.001**
lh precuneus thickness	1.95	0.24	1.89	0.26	2.23	0.15	**0.012**	**< 0.001**
lh rostralanteriorcingulate thickness	2.39	0.32	2.55	0.41	2.80	0.25	**0.014**	**0.049**
lh rostralmiddlefrontal thickness	2.01	0.25	2.07	0.24	2.27	0.11	**0.017**	**0.002**
lh superiorfrontal thickness	2.29	0.31	2.26	0.28	2.54	0.17	**0.049**	**< 0.001**
lh superiorparietal thickness	1.85	0.22	1.75	0.24	2.08	0.14	**0.008**	**< 0.001**
lh superiortemporal thickness	1.86	0.26	2.17	0.31	2.57	0.17	**< 0.001**	**< 0.001**
lh supramarginal thickness	2.13	0.24	1.97	0.22	2.38	0.13	**0.025**	**< 0.001**
lh frontalpole thickness	2.38	0.23	2.56	0.39	2.72	0.25	**0.018**	0.372
lh temporalpole thickness	2.34	0.51	3.06	0.59	3.47	0.39	**< 0.001**	**0.037**
lh transversetemporal thickness	1.97	0.37	1.92	0.34	2.21	0.25	0.191	**0.008**
lh insula thickness	2.51	0.29	2.55	0.37	2.91	0.21	**0.012**	**< 0.001**
rh bankssts thickness	2.06	0.27	2.12	0.23	2.44	0.16	**0.005**	**< 0.001**
rh caudalanteriorcingulate thickness	2.53	0.14	2.57	0.28	2.63	0.28	0.343	0.654
rh caudalmiddlefrontal thickness	2.16	0.32	2.18	0.16	2.38	0.12	0.059	**< 0.001**
rh cuneus thickness	1.64	0.15	1.66	0.13	1.75	0.20	0.211	0.063
rh entorhinal thickness	2.28	0.32	2.83	0.47	3.38	0.40	**< 0.001**	**0.002**
rh fusiform thickness	2.15	0.22	2.30	0.18	2.57	0.11	**< 0.001**	**< 0.001**
rh inferiorparietal thickness	2.09	0.20	2.01	0.20	2.32	0.14	**0.017**	**< 0.001**
rh inferiortemporal thickness	2.36	0.25	2.43	0.23	2.70	0.17	**0.008**	**< 0.001**
rh isthmuscingulate thickness	2.11	0.13	1.99	0.16	2.26	0.17	0.092	**< 0.001**
rh lateraloccipital thickness	1.97	0.20	1.96	0.14	2.09	0.16	0.220	**0.005**
rh lateralorbitofrontal thickness	2.38	0.19	2.51	0.22	2.52	0.16	0.103	0.873
rh lingual thickness	1.82	0.14	1.81	0.10	1.90	0.16	0.234	**0.037**
rh medialorbitofrontal thickness	2.28	0.21	2.45	0.19	2.41	0.19	0.199	0.524
rh middletemporal thickness	2.28	0.23	2.48	0.22	2.72	0.17	**0.005**	**< 0.001**
rh parahippocampal thickness	2.01	0.30	2.31	0.38	2.51	0.24	**0.010**	0.094
rh paracentral thickness	2.15	0.19	2.03	0.21	2.23	0.23	0.338	**0.005**
rh parsopercularis thickness	2.22	0.29	2.29	0.19	2.46	0.12	**0.043**	**0.005**

rh parsorbitalis thickness	2.45	0.18	2.48	0.20	2.52	0.18	0.477	0.524
rh parstriangularis thickness	2.18	0.21	2.25	0.16	2.31	0.10	0.243	0.198
rh pericalcarine thickness	1.50	0.17	1.50	0.18	1.57	0.18	0.350	0.179
rh postcentral thickness	1.81	0.17	1.82	0.13	1.97	0.18	0.059	0.002
rh posteriorcingulate thickness	2.24	0.18	2.19	0.18	2.38	0.19	0.112	0.008
rh precentral thickness	2.20	0.27	2.11	0.26	2.35	0.23	0.220	0.002
rh precuneus thickness	2.00	0.23	1.99	0.19	2.18	0.17	0.065	0.007
rh rostralanteriorcingulate thickness	2.62	0.26	2.76	0.25	2.77	0.26	0.243	0.847
rh rostralmiddlefrontal thickness	2.09	0.22	2.15	0.13	2.25	0.12	0.061	0.044
rh superiorfrontal thickness	2.33	0.26	2.35	0.14	2.52	0.14	0.070	< 0.001
rh superiorparietal thickness	1.92	0.24	1.84	0.16	2.07	0.16	0.092	< 0.001
rh superiortemporal thickness	2.16	0.26	2.34	0.21	2.60	0.19	**0.005**	**< 0.001**
rh supramarginal thickness	2.21	0.16	2.08	0.12	2.36	0.17	0.050	**< 0.001**
rh frontalpole thickness	2.53	0.21	2.70	0.23	2.76	0.29	0.071	0.461
rh temporalpole thickness	2.83	0.72	3.17	0.53	3.59	0.35	**0.012**	**0.008**
rh transversetemporal thickness	2.10	0.46	2.16	0.22	2.26	0.25	0.420	0.094
rh insula thickness	2.69	0.13	2.68	0.29	2.92	0.20	**0.016**	**0.002**
